# Hydroxyapatite Obtained via the Wet Precipitation Method and PVP/PVA Matrix as Components of Polymer-Ceramic Composites for Biomedical Applications

**DOI:** 10.3390/molecules26144268

**Published:** 2021-07-14

**Authors:** Magdalena Głąb, Sonia Kudłacik-Kramarczyk, Anna Drabczyk, Janusz Walter, Aleksandra Kordyka, Marcin Godzierz, Rafał Bogucki, Bożena Tyliszczak, Agnieszka Sobczak-Kupiec

**Affiliations:** 1 Department of Materials Science, Faculty of Materials Engineering and Physics, Cracow University of Technology, 37 Jana Pawła II Av., 31-864 Krakow, Poland; janusz.walter@pk.edu.pl (J.W.); rafal.bogucki@pk.edu.pl (R.B.); bozena.tyliszczak@pk.edu.pl (B.T.); agnieszka.sobczak-kupiec@pk.edu.pl (A.S.-K.); 2 Centre of Polymer and Carbon Materials Polish Academy of Sciences, M. Curie-Skłodowskiej 34 St., 41-819 Zabrze, Poland; akordyka@cmpw-pan.edu.pl (A.K.); mgodzierz@cmpw-pan.edu.pl (M.G.)

**Keywords:** hydroxyapatite, polymer-ceramic composites, PVP/PVA-based polymers, sedimentation rate, sorption capability, tissue engineering

## Abstract

The aspect of drug delivery is significant in many biomedical subareas including tissue engineering. Many studies are being performed to develop composites with application potential for bone tissue regeneration which at the same provide adequate conditions for osteointegration and deliver the active substance conducive to the healing process. Hydroxyapatite shows a great potential in this field due to its osteoinductive and osteoconductive properties. In the paper, hydroxyapatite synthesis via the wet precipitation method and its further use as a ceramic phase of polymer–ceramic composites based on PVP/PVA have been presented. Firstly, the sedimentation rate of hydroxyapatite in PVP solutions has been determined, which allowed us to select a 15% PVP solution (sedimentation rate was 0.0292 mm/min) as adequate for preparation of homogenous reaction mixture treated subsequently with UV radiation. Both FT-IR spectroscopy and EDS analysis allowed us to confirm the presence of both polymer and ceramic phase in composites. Materials containing hydroxyapatite showed corrugated and well-developed surface. Composites exhibited swelling properties (hydroxyapatite reduced this property by 25%) in simulated physiological fluids, which make them useful in drug delivery (swelling proceeds parallel to the drug release). The short synthesis time, possibility of preparation of composites with desired shapes and sizes and determined physicochemical properties make the composites very promising for biomedical purposes.

## 1. Introduction

Bone surgery and tissue engineering are currently some of the most rapidly developing fields of medicine. One of the main purposes of these medical disciplines is bone defect treatment. Bone defects may be caused mainly by congenital anomalies, trauma, infections or cancer diseases (when tissue resection is necessary) [1,2]. Other factors affecting the loss of bone tissue density or mass are traffic accidents, old age and sport-related injuries such as fractures [3]. The most popular approach for bone defect repair is surgical treatment via autologous or artificial bone grafting. Nonetheless, these methods are related to the occurrence of such complications as, e.g., strong pain in the site of the surgery or transmission of various pathogens, which may contribute to additional health problems such as sepsis. Thus, alternative methods are being sought and one of them seems to be an application of synthetic materials with might affect the osteoblasts to differentiate and form new tissue which will fill the defect [4].

A promising material with great application potential for medical purposes, including tissue regeneration, implantology or orthopedics, is hydroxyapatite [5]. Hydroxyapatite (HAp) is a main inorganic component of vertebrate teeth and bones. It was proven that it shows bioactivity, biocompatibility and non-immunogenicity [6]. Importantly, HAp is osteoconductive, i.e., it shows the capability of forming a material on which the cells are able to grow, and is osteoinductive, i.e., it induces the osteogenesis process [7,8]. Importantly, hydroxyapatite provides also a good osteointegration so it allows formation of the direct functional and structural connection between the tissue and the biomaterial modified with this inorganic compound [9,10,11]. Due to these features HAp is often used currently for preparation of various biomaterials [12,13]. For example, a growing interest is currently observed for application of hydroxyapatite in preparation of coatings for implants. Such an application was proposed, e.g., by Abdal-hay et al. [14], Sutha et al. [15] and Kim et al. [16]. In turn, Goranova et al. proposed hydroxyapatite as one of the components of nanografts designed for use as bone cement additives [17].

Many investigations are currently being performed to develop polymer–ceramic scaffolds containing hydroxyapatite that may fill the bone defect and form conditions adequate for bone repair. Studies on the development of bone scaffolds consisting of carbon-based nanomaterials, hydroxyapatite and polyetheretherketone (PEEK) were described by Swaminathan et al. They proved that the developed bionanocomposites showed high porosity, non-toxicity and good cell viability [18]. In turn, scaffolds containing non-organic silica, chitosan and hydroxyapatite were obtained and characterized by Adamski and Siuta. They proved the high porosity of prepared materials, non-toxicity towards fibroblasts and the density comparable to the density of natural bone [19]. Next, chitosan, hydroxyapatite and poly(vinyl alcohol) were used for synthesis of polymer-ceramic composites by Pineda-Castillo et al. The materials were defined as adequate for colonization, differentiation and proliferation of osteoblasts [20]. For the preparation of polymer matrices for hydroxyapatite, the use of such polymers or co-polymers as poly(lactic-co-glycolic acid) [21,22], poly(trimethylene carbonate) [23], polycaprolactone [24,25] or polylactide [26] was also reported. Importantly, apart from the previously mentioned chitosan, other biopolymers may be also used for the preparation of such scaffolds, e.g., cellulose [27] or collagen [28].

The combination of various polymers with the ceramic phase leads to the preparation of composite materials with desired mechanical properties. The ceramic phase corresponds to the adequate mechanical strength and rigidity of such composite wherein the polymer phase acts as a biocompatible and non-toxic matrix providing the appropriate elasticity of such formed material. Furthermore, the ceramic phase in a form of a hydroxyapatite provides bioactive properties and affects the bone tissue regeneration [29]. Despite of the fact that composite materials show many advantages, the possibility of the implant rejection due to the numerous bacterial infections still constitutes a problematic issue [30]. One of the most promising solutions seems to be the development of the composite materials modified with appropriate substances such as, e.g., silver nanoparticles showing antibacterial properties [31].

The main purpose of the research presented in this paper was to obtain polymer-ceramic composites with application potential for bone tissue regeneration. The novelty of the work involved the combination of poly(vinyl alcohol) (PVA) and polyvinylpyrrolidone (PVP) as a polymer matrix for hydroxyapatite and synthesis of such composites using UV radiation. Firstly, hydroxyapatite powder was prepared via the wet precipitation method. Next, the crystallinity of the powder obtained was characterized by X-ray diffraction (XRD) technique. An important aspect of the research was to evaluate the sedimentation rates of hydroxyapatite in PVP solutions at various concentrations to select adequate solution for preparation of the reaction mixture treated subsequently with UV radiation. The chemical structure of composites was characterized by FT-IR spectroscopy and their surface morphology was analyzed using SEM technique wherein the imaging was additionally accompanied by the elemental analysis. Moreover, swelling properties of the composites were also evaluated.

## 2. Results and Discussion

### 2.1. Results of XRD Analysis Performed to Characterize the Crystallinity of HAp Powder Prepared

**Figure 1:**
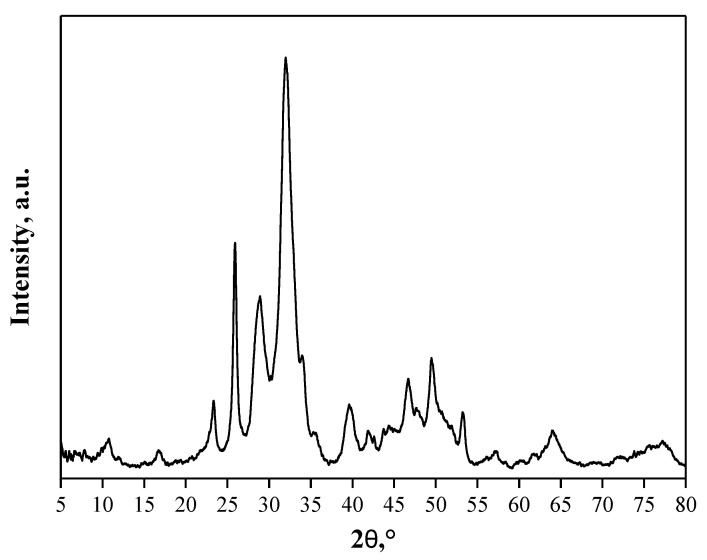
X-ray diffraction pattern of synthesized HAp.

The diffractogram obtained is presented in Figure 1. Additionally, the data obtained as a result of the performed investigation is reported in Table 1.

Figure 1 presents the diffractogram of obtained material. Using ICDD database two phases were identified: hydroxyapatite (ICDD # 00–064–0738) and tricalcium phosphate (TCP) (ICDD # 00–029–0359). HAp crystals have the space group P6_3_/m with a hexagonal crystal structure, while TCP is represented by the monoclinic P2_1_/a space group. The unit cell parameters for both phases are similar to those given in the ICDD. In the case of HAp, unit cell dimensions increased slightly. For TCP, the parameters a, c, and the β angle slightly increased, while b decreased. Crystal sizes for HAp are about 7.8 nm and for TCP about 17 nm.

### 2.2. Studies on the Hydroxyapatite Suspension Stability

**Figure 2:**
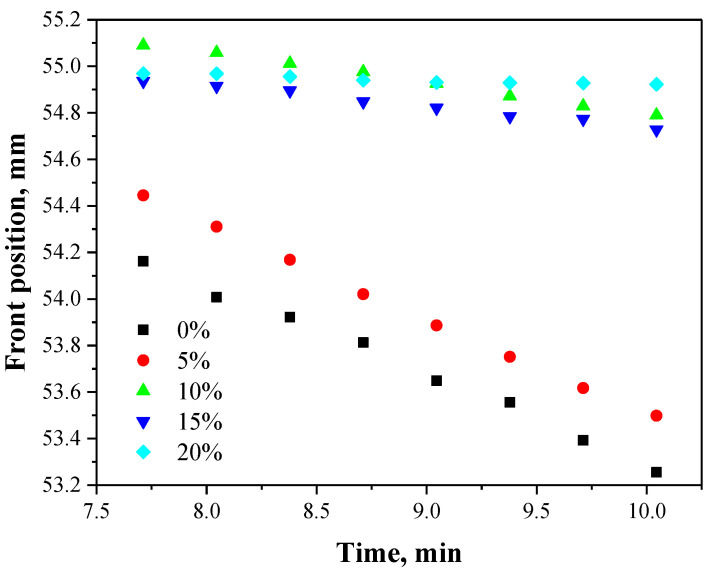
Migration front as a function of time.

In order to select the adequate concentration of the PVP solution used for the synthesis of polymer–ceramic composites, the stability of the ceramic phase (HAp) in the polymer solutions at various concentrations was evaluated. Among the analyzed concentrations—i.e., 0%, 5%, 10%, 15%, 20% and 25%—the highest one was not considered for further syntheses due to the fact that such a solution was not homogeneous. In the case of the other concentrations, the stability evaluation by determining the migration front as a function of time was performed. The results obtained are shown in Figure 2.

As it may be seen above, the results obtained show the differences between the stability of tested hydroxyapatite suspensions. Based on the different course of the migration fronts, it may be indicated that the concentration of the PVP solution affected the observed sedimentation process. It is possible to notice in Figure 2 that the stable migration front was reported for HAp suspension in 20% PVP solution. In the case of samples named as “0%” and “5%” the front position decreased slightly in time (HAp particles in the suspension moved slightly towards the bottom of the vessel in which the study was conducted). For the concentrations 10% and 15%, the changes in front positions were also observed but much smaller than in the case of the concentration 5% and 0%. For example, the migration front position of the sample with 5% PVP solution fluctuated within the range 53.49–54.45 while for the sample with 15% PVP solution, the range of the measured parameter was significantly lower, i.e., 54.72–54.93. Below in Table 2, the sedimentation rates for HAp particles depending on the concentration of PVP solutions used are presented.

**Figure 3:**
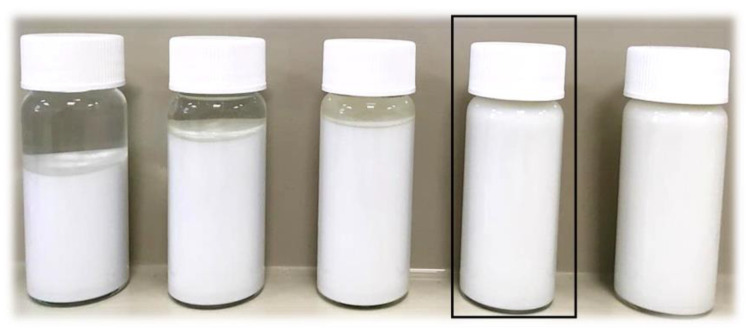
The HAp suspensions in PVP solutions directly after the study (from left: 0%, 5%, 10%, 15% and 20% PVP).

In Figure 3, images of tested HAp suspensions directly after the measurements reflecting the differences between the sedimentation rates of particular samples are shown.

Many factors have the impact on the sedimentation process. Some of the most important ones are the size of the particles dispersed in the suspension and the viscosity of the liquid, which determines the friction between the particles and the liquid [32]. The highest sedimentation rate was reported for sample named as “0%” so for the suspension of hydroxyapatite in distilled water. Next, it was observed that as the PVP solution concentration increased, the sedimentation rate decreased. This may be related to the fact that as the polymer solution concentration increased, its viscosity also increased.

During the preparation of polymer-ceramic composites a very important aspect is to obtain a stable and relatively homogeneous suspension of the reagents used. Thus, in the case of samples consisting of 15% and 20% of PVP solutions their sedimentation rate is too low (0.0292 mm/min and 0.0161 mm/min, respectively). The polymerization process leading to the synthesis of composites takes approximately 120 s. Therefore, it is assumed that the sedimentation rates for samples with 15% and 20% solutions are appropriate for preparation of the homogeneous solution. It is also assumed that the crosslinking of the reaction mixture will take place before the rapid sedimentation of the ceramic phase. Importantly, considering the principles of “green chemistry” as well as the economic aspect concerning the reducing the use of non-renewable substrates [33]. The 15% PVP solution was selected for the synthesis of polymer-ceramic composites (marked with a black frame in Figure 3). The solution with such a concentration allowed us to prepare an adequately stable suspension of HAp particles, which is desirable in viewpoint of the photopolymerization process.

### 2.3. Results of the Analysis of the Structure of the Composites via FT-IR Technique

**Figure 4:**
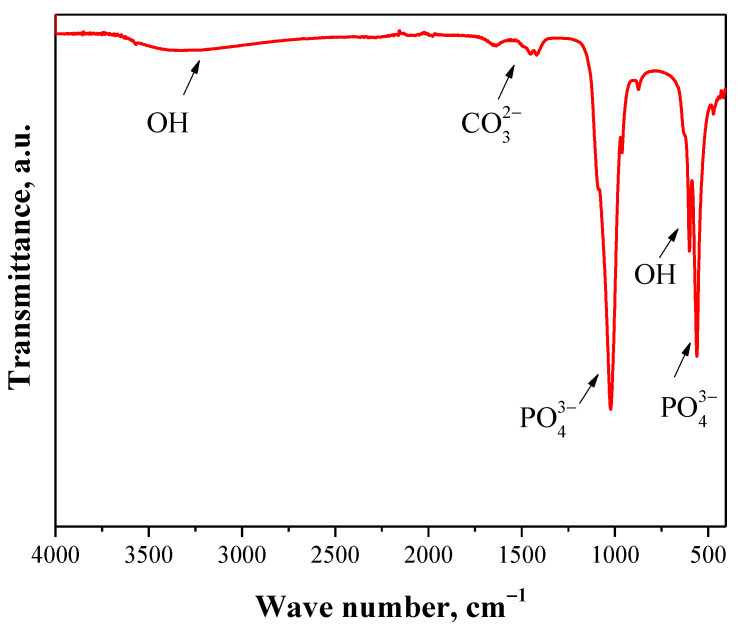
FT-IR spectrum of hydroxyapatite.

**Figure 5:**
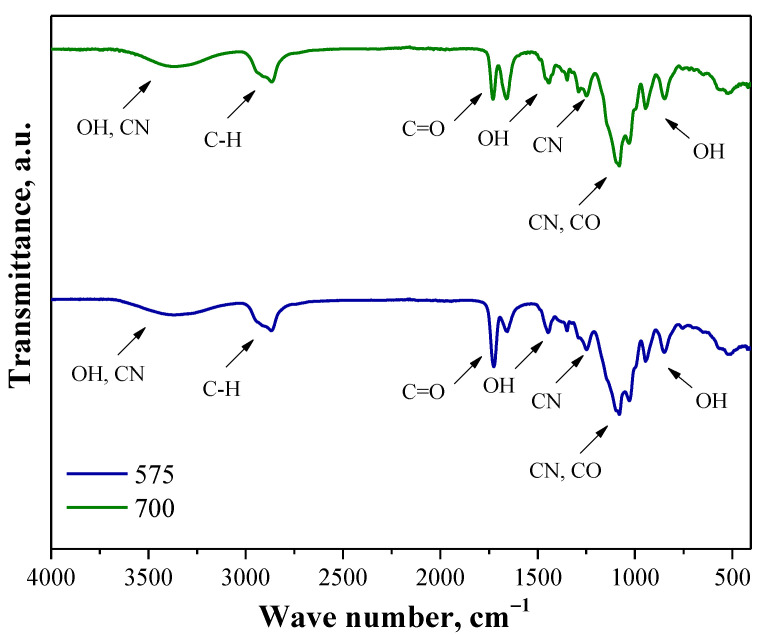
FT-IR spectra of polymer matrices obtained using crosslinker with average molecular weight 575 g/mol (blue) and 700 g/mol (green).

**Figure 6:**
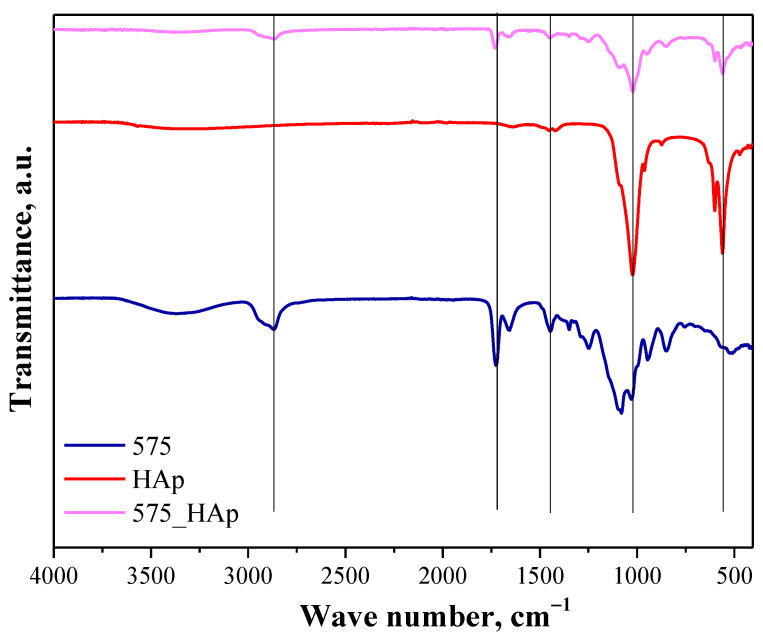
FT-IR spectrum of HAp (red), polymer matrix obtained using PEGDA 575 (575, blue) and the composite obtained using the same crosslinker (575_HAp; pink).

**Figure 7:**
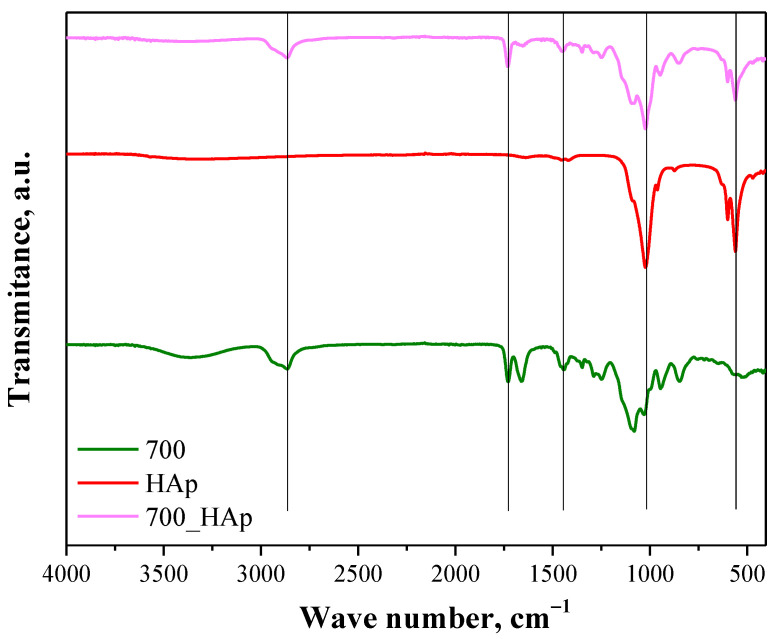
FT-IR spectrum of HAp (red), polymer matrix obtained using PEGDA 700 (700, green) and the composite obtained using the same crosslinker (700_HAp; pink).

FT-IR analysis was performed for hydroxyapatite, polymer matrices and final polymer-ceramic composites prepared using crosslinkers with various molecular weights. FT-IR spectra obtained are presented in Figure 4, Figure 5, Figure 6 and Figure 7.

In Figure 4, FT-IR spectrum of prepared hydroxyapatite is shown. The wide absorption band of relatively low intensity within the range 3550 cm^−1^–2975 cm^−1^ observed on the FT-IR spectrum may be assigned to the valence vibrations of OH group [34]. Moreover, the absorption band visible at 620 cm^−1^ corresponds also to the hydroxyl group [34]. Next, the band at 1425 cm^−1^ indicates the presence of asymmetric stretching vibrations deriving from carbon ions [35]. At 1025 cm^−1^ and 560 cm^−1^ absorption bands corresponding to the phosphate groups—which are characteristic for hydroxyapatite—may also be noticed, wherein the first mentioned band may be attributed to the stretching vibrations of O-P-O bond, while the second one corresponds to the triply degenerated bending mode of -O-P-O present in the PO_4_^3−^ groups [36].

Next, in Figure 5 the FT-IR spectra of polymer matrices prepared using various crosslinking agents are shown.

The spectra presented in Figure 5 derive from polymer matrix wherein the difference between these matrices is the molecular weight of the crosslinker used for its preparation. On both spectra the adsorption bands at analogous wavenumber ranges may be observed. These bands are the overlapping bands corresponding to the functional groups of polymers used for the synthesis of the tested matrices, i.e., PVA and PVP. The wide band of a low intensity observed between 3566 cm^−1^ and 3150 cm^−1^ is characteristic for intermolecular and intramolecular hydrogen bonds occurring between the OH groups in the structure of PVA [37]. Nonetheless, these bands may also overlap with bands characteristic for stretching vibrations of CN group deriving from PVP [38]. Next, the absorption band at a wavenumber of 2910 cm^−1^ may be assigned to the stretching vibrations of C-H bond characteristic for alkyl group of both applied polymers [37]. In turn, the bands at 1660 cm^−1^ and 1723 cm^−1^ are also characteristic for both polymers and may be attributed to the stretching vibrations of CO group [39,40]. Next, a band at 1441 cm^−1^ deriving from the inner-plane bending oscillations of OH group may also be reported [41]. Moreover, the absorption band corresponding to the stretching vibrations of CN group of PVP (at 1254 cm^−1^) as well as overlapping bands deriving from CN and CO groups (at 1083 cm^−1^) from this polymer may also be noticed [38]. In turn, the band visible within the range 940 cm^−1^–835 cm^−1^, which may be attributed to the out-of-plane bending vibrations of hydroxyl group was also observed [41].

Below in Figure 6 and Figure 7, the next FT-IR spectra are shown. They are presented in such a way to indicate the differences between polymer matrix and polymer-ceramic composite prepared using the same crosslinker (having the same molecular weight). Additionally, FT-IR spectrum of hydroxyapatite was also shown in both figures to enable the indication of the bands deriving from this material both in the spectrum of polymer matrix and composite.

The main attention was paid to the comparison of the spectra of composites with the spectra of hydroxyapatite and polymer matrix without this additive. Both in Figure 6 and Figure 7, in the case of the spectra of composites, the absorption bands at approx. 561 cm^−1^ and 1023 cm^−1^ correspond and overlap with the absorption bands of phosphate groups of hydroxyapatite. In turn, the absorption bands at 1440 cm^−1^, 1723 cm^−1^ and 2909 cm^−1^ overlap with bands characteristic for functional groups occurring in the structure of components forming the polymer matrix. All overlapping bands are marked with black lines. FT-IR analysis allowed us to confirm the presence of both the polymer phase and the ceramic phase in obtained composites.

### 2.4. Results of SEM-EDS Analysis

**Figure 8:**
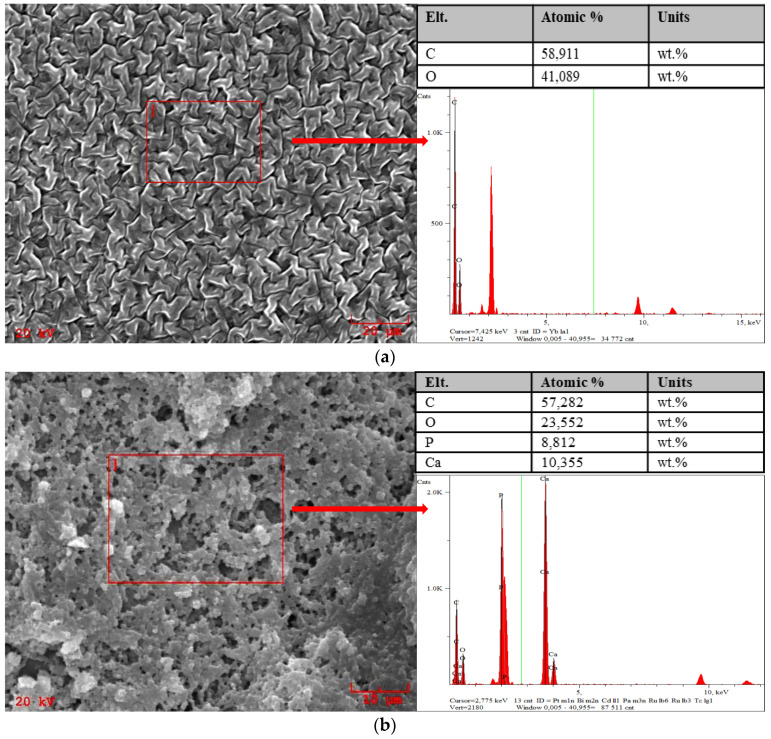

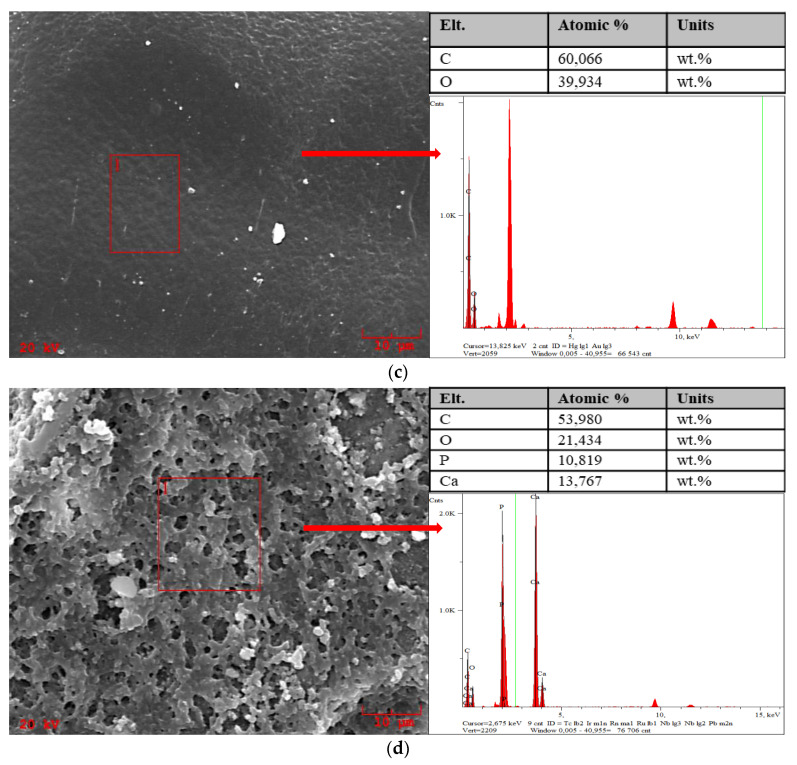
SEM-EDS analysis of sample 575 (**a**), 575_HAp (**b**), 700 (**c**) and 700_HAp (**d**).

The surface morphology of both polymer matrices and polymer–ceramic composites was characterized via the SEM technique. The performed SEM analysis also included an elemental analysis. Obtained images and corresponding point elemental analyses are presented below in Figure 8.

Considering the results of SEM imaging of polymer matrices, it may be concluded that the molecular weight used for their synthesis affected the surface morphology of obtained materials. Surfaces of sample 575 and sample 700 differ significantly. It may be observed that the surface of sample obtained using PEGDA 575 shows a significantly more developed structure. Moreover, it is more corrugated than the surface of sample 700, which is relatively smooth. This is due to the use of crosslinker with lower molecular weight. As a result of the crosslinking process, a formation of short polymer chains is observed. Thus, the sample obtained has a tightly packed structure and its surface is characterized by a greater corrugation compared to the surface of sample prepared using crosslinker with higher molecular weight. In the case of the use of PEGDA 700 during the synthesis, the polymer chains in the structure of crosslinked material are longer thus the structure of such formed polymer is smoother.

Comparing the surface morphology of both analyzed polymer-ceramic composites—i.e., sample 575_HAp and sample 700_HAp—no significant changes were observed. Nonetheless, the differences between the polymer matrices and the composites obtained using the same crosslinker are clearly visible. The presence of the ceramic phase may be observed. Furthermore, the clear advantage is preparation of the materials with a relatively homogeneous surface which is the result of its complete coverage by the HAp particles. It may be also concluded that the obtained polymer–ceramic composites show a porous and well-developed specific surface area, which is a significant advantage of the developed materials considering their potential application for bone tissue regeneration accompanied by the cell overgrowth.

In turn, based on the results of the elemental analysis accompanying the SEM images it may be concluded that in the case of both tested polymer matrices the only elements identified were carbon and oxygen so the main elements of reagents forming these matrices. In the case of the polymer–ceramic composites the presence of calcium and phosphorus was proved thus confirming the presence of the hydroxyapatite in the tested materials. Such results are consistent with the results of the FT-IR spectroscopy and also indicate the occurrence of both polymer and ceramic phase in obtained composite materials.

### 2.5. Results of Studies on Swelling Properties

**Figure 9:**
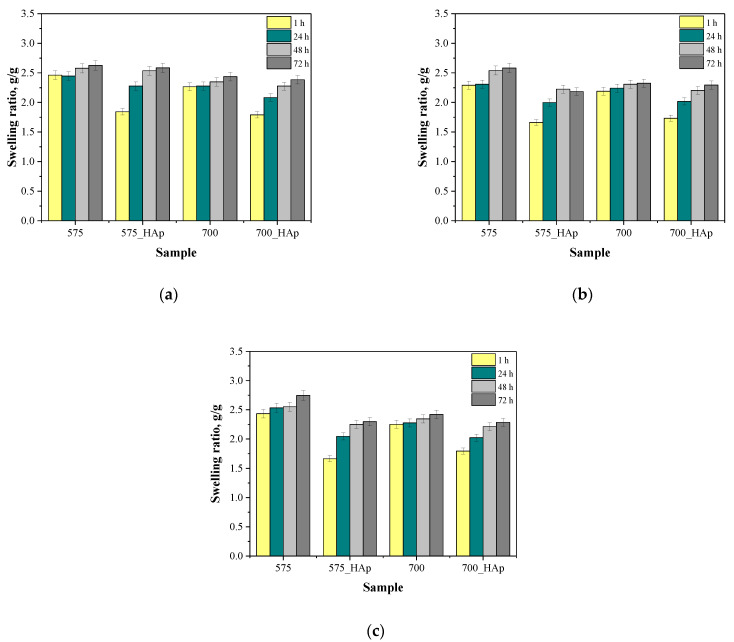
Results of sorption analysis in distilled water (**a**), SBF (**b**) and Ringer liquid (**c**).

In Figure 9, the results of the investigations on the sorption properties of prepared polymer-ceramic composites are shown. Results are presented in a form of bar charts showing the swelling sorption of samples depending on their composition and the liquid in which the study was performed.

Based on the performed investigations it was proved that all tested composites showed swelling properties. The calculated values of swelling ratios depended both on the composition of the tested sample and on the type of the swelling medium. The highest differences between the swelling ratios of composites obtained using crosslinkers with different molecular weights were observed during the first 24 h of the study. It was observed that the use of PEGDA 575 leaded to the preparation of polymer matrix consisting of shorter polymer chains compared to these ones forming the polymer matrix synthesized using PEGDA 700. Moreover, the surface of this material is porous and corrugated, which was confirmed via the analysis performed using scanning electron microscope. Thus at the beginning of the sorption study—i.e., in the first hours of the study—while the surface sorption of liquids takes place, the differences between the values of swelling ratios calculated for samples obtained using PEGDA 575 and PEGDA 700 are the most noticeable. During the further course of the research, i.e., after 48 and 72 h, the differences in swelling ability of tested samples were slight, which may be due to the penetration of the liquid absorbed into the material.

On the other hand, polymer matrices obtained using PEGDA 700 consisted of long polymer chains. As a result, the functional groups occurring in the structure of PVA and PVP responsible for interactions with absorbed liquid may be embedded between these chains. In the case of samples prepared using PEGDA 575 these groups were probably more exposed and thus more available for interactions with absorbed liquid (such as, hydrogen bonds) which, in turn, reflected in higher swelling sorption of such material.

Analyzing the sorption properties of samples in viewpoint of the swelling medium used it was proved that the highest swelling ratios were calculated for samples swelling in distilled water. In the case of SBF and Ringer liquid a significantly lower sorption capability was reported, which is probably related to the chemical compositions of these liquids. For example, divalent calcium ions present in both solutions may contribute to the formation of additional crosslinks in the polymer matrix increasing the crosslinking density of such material and decreasing free spaces for absorbed liquids. 

Additional differences may be observed in the case of samples containing ceramic phase. The swelling ratios of composites with hydroxyapatite were lower compared to analogous unmodified polymer matrices. This, in turn, may be related to the fact that the ceramic phase fills free spaces between polymer chains decreasing in such a way the volume of free spaces available for absorbed liquids.

## 3. Materials and Methods

### 3.1. Materials

Ammonium phosphate monobasic (NH_4_H_2_PO_4_, ACS reagent, ≥98%), calcium nitrate tetrahydrate (Ca(NO_3_)_2_∙4H_2_O, ACS reagent, 99%) and ammonia water (NH_4_OH, 25%) used for the synthesis of hydroxyapatite as well as diacrylate poly(ethylene glycol) (crosslinking agent, average molecular weight M_n_ = 700 g/mol (PEGDA 700) and M_n_ = 575 g/mol (PEGDA 575)); 2-hydroxy-2-methylpropiophenone (photoinitiator, 97%, d = 1.077 g/mL), poly(vinyl alcohol) (PVA, M_w_ = 13,000–23,000 g/mol; 87–89%; hydrolyzed, crystalline powder) and polyvinylpyrrolidone (PVP, average molecular weight 10,000 g/mol, powder) used for the synthesis of composites were bought from Sigma Aldrich (Saint Louis, MO, USA). All reagents were applied as obtained without further purification.

### 3.2. Synthesis of Hydroxyapatite (HAp)

**Figure 10:**
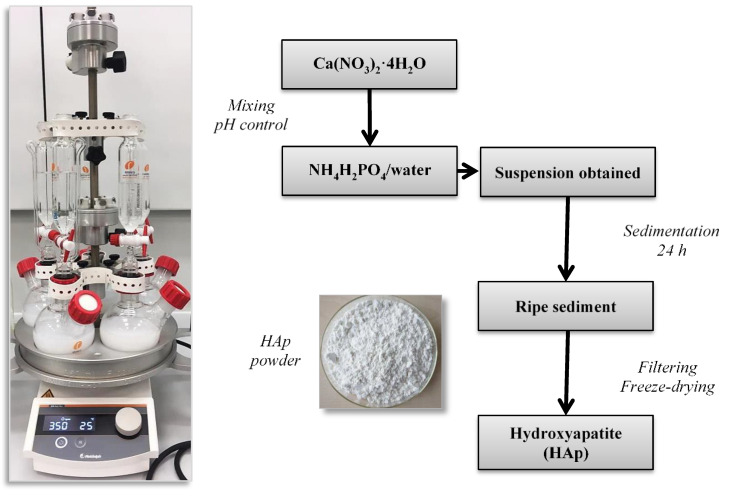
The equipment used (left) and the scheme (right) of the preparation of hydroxyapatite.

Hydroxyapatite was obtained via the wet precipitation method wherein the ammonium phosphate monobasic and calcium nitrate tetrahydrate were used as the main reagents. In the first stage, 20 mL of NH_4_H_2_PO_4_ solution (0.36 mol/L) were introduced into the flasks in Carousel Plus Reaction Station (Radleys, United Kingdom) containing 130 mL of distilled water. Then, the ammonia water was added until the pH of the reaction mixture was >10. Next, 50 mL of Ca(NO_3_)_2_∙4H_2_O (0.60 mol/L) was added dropwise (1 drop/s) to the mixture. Process was performed at constant stirring (350 rpm) and at 25 °C. After adding the whole volume of the calcium nitrate solution, the reaction mixture was mixed for further 30 min and remained for 24 h. Then, the ripening and the sedimentation of the precipitate formed took place. After 24 h, the precipitate was filtrated and washed with distilled water to the neutral pH. The equipment applied as well as the scheme of the hydroxyapatite synthesis is presented in Figure 10.

The hydroxyapatite obtained has been investigated via XRD technique and FT-IR analysis. Next, its stability in PVP solutions at various concentrations has also been determined. In further step of the research, the HAp powder prepared was used for preparation of polymer-ceramic composites.

### 3.3. Synthesis of Polymer-Ceramic Composites

In the first step of the performed works, the main attention was to select adequate concentration of PVP solution used as one of the main components of the developed composites. The analysis of the stability of HAp suspensions in various PVP solutions enabled to select adequate concentration of PVP solution used for the preparation of the composites, i.e., 15% (discussion over the results of stability measurements is presented in Section 3.2 of the paper). Subsequently, the PVP solution was mixed with 5% PVA solution and the adequate amounts of the ceramic phase—hydroxyapatite—was obtained. Finally, the PEGDA 575 as crosslinking agent and the 2-hydroxy-2-methylpropiophenone as photoinitiator were introduced into the reaction mixture. After the intensive stirring the prepared mixtures were poured down into the vessels and subjected to the photopolymerization. The process was performed for 120 s using EMITA VP-60 lamp (power: 180 W, λ = 320 nm). 

Further works included the syntheses performed in the same manner (the same conditions and the same amounts of the reagents were used) wherein using crosslinking agent with higher molecular weight (PEGDA 700) and without the addition of hydroxyapatite. The amounts of the reagents used for preparation of all synthesized composites are presented in Table 3.

**Figure 11:**
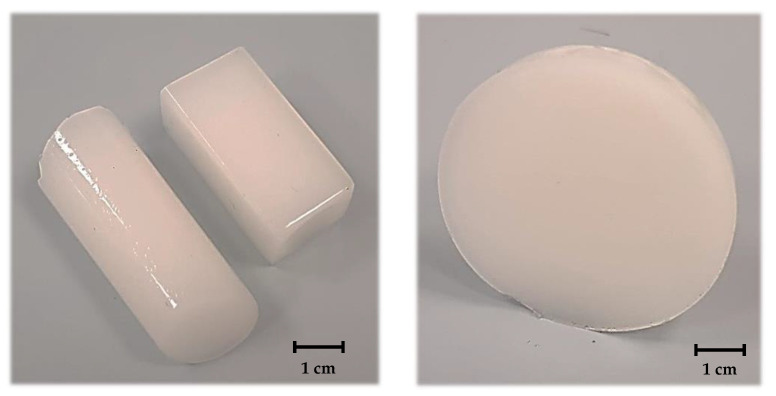
Various shapes of prepared ceramic-polymer composites.

The proposed methodology allowed us to prepare composites having various shapes and sizes which constitutes their big advantage in viewpoint of their potential application for bone tissue regeneration. This is because it will be possible to adjust the adequate reaction vessel to obtain material suitable for specific bone cavity. Example images of obtained composite materials are presented in Figure 11.

The obtained composites were characterized using techniques such as XRD (X-ray diffraction), FT-IR spectroscopy (Fourier transform infrared spectroscopy) and SEM-EDS (Scanning Electron Microscopy-Energy Dispersive X-ray Spectroscopy). Moreover, the stability of the suspension of the ceramic phase in the polyvinylpyrrolidone (PVP) solution was verified using Multiscan MS 20 equipment. Additionally, the sorption properties of obtained composites in selected fluids (distilled water, simulated body fluid and Ringer liquid) were also evaluated.

### 3.4. Methodology of Performed Investigations

#### 3.4.1. Analysis of Crystallinity of HAp Powder Obtained via X-ray Diffraction (XRD) Technique

XRD measurements were performed using the D8 Advance diffractometer (Bruker, Karlsruhe, Germany) with Cu-Kα cathode (λ = 1.54 Å). The scan rate was 4.8°/min with scanning step 0.02**°** in range of 5° to 80° 2Θ, using Bragg–Brentano geometry. Identification of fitting phases was performed using DIFFRAC.EVA program with ICDD database. Lattice parameters, strains and crystal sizes were calculated using Rietveld refinement in TOPAS 6 program, basing on Williamson–Hall theory. The pseudo-Voigt function was used in the description of diffraction line profiles at the Rietveld refinement. The R_wp_ (weighted-pattern factor) and S (goodness-of-fit) parameters were used as numerical criteria of the quality of the fit of calculated to experimental diffraction data.

#### 3.4.2. Evaluation of the Stability of Hydroxyapatite Suspensions

**Figure 12:**
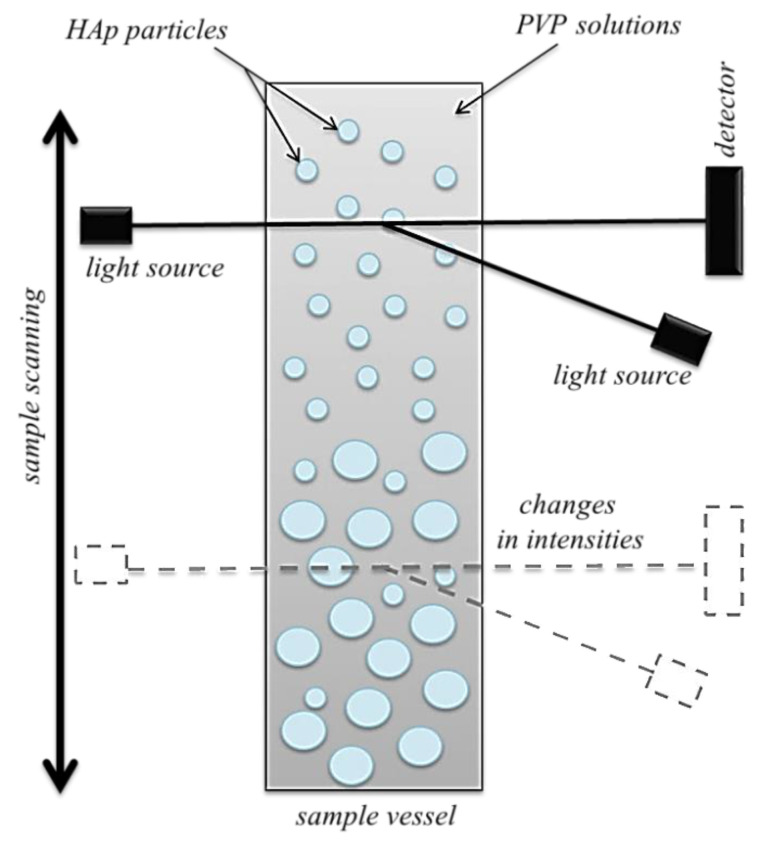
The scheme of the investigations on the stability of HAp suspension in PVP solution.

In order to select adequate concentration of PVP solution used for the synthesis of composites, studies on the stability of the suspension of ceramic phase (hydroxyapatite) in the solutions of this polymer at various concentrations were conducted. The following concentrations of PVP solutions were proposed: 5%, 10%, 15%, 20% and 25%, respectively. Furthermore, the stability of hydroxyapatite in distilled water (reference measurement defined as “0% PVP”) was also determined. The stability of tested samples was measured via system of the stability analysis—MultiScan MS20 DataPhysics Instruments (Charlotte, NC, USA). The sedimentation rate of hydroxyapatite in tested suspensions was determined based on the transmission and the backscattering analysis. The scheme of the research is shown in Figure 12.

Measurements were performed at 25 °C. In the case of all samples, the study was conducted for 10 min (1 scan/20 s).

#### 3.4.3. Characterization of Obtained Materials via Fourier Transform Infrared (FT-IR) Spectroscopy

FT-IR analysis was performed to verify the functional groups present in the structure of the tested composites. HAp powder, polymer matrices and composites containing 10% of the ceramic phase were subjected to the measurements. These were conducted using the Thermo Scientific Nicolet iS5 FT-IR spectrophotometer equipped with iD7 ATR (Loughborough, UK). FT-IR spectra were recorded at room temperature and within the wavelength range 4000–400 cm^−1^.

#### 3.4.4. SEM-EDS Analysis of Prepared Materials

SEM-EDS (Scanning Electron Microscopy–Energy Dispersive X-ray spectroscopy) technique was used to characterize the surface morphology of polymer matrices and HAp/PVP/PVA composites. The study was carried out using Jeol 5510LV Scanning Electron Microscope equipped with EDS IXRF System detector (Freising, Germany). EDS system enabled to define the elemental composition of the top layer of analyzed materials. Before the measurements, samples were dried and subjected to the gold sputtering to provide the adequate conductivity of tested materials.

#### 3.4.5. Evaluation of the Sorption Properties of the Developed Materials 

The main goal of the next investigations was to assess the sorption properties of the developed materials. The study was conducted for polymer matrices obtained both with crosslinker with an average molecular weight 575 g/mol and 700 g/mol as well as for polymer–ceramic composites. For this purpose, the obtained samples were weighed (approximately 1.0 g each) and placed in 50 mL of the adequate liquid, wherein the study was performed using distilled water, simulated body fluid (SBF) and Ringer liquid (fluid isotonic to the human blood). After a certain period of time—i.e., 1 h, 24 h and 48 h—samples were separated from the liquid, an excess free (unbound) water was removed from the surfaces of the swollen materials and then they were weighed again. The swelling ability of analyzed samples was determined using the swelling ratio (*α*) calculated based on the following Equation (1):

(1) $$\alpha =\frac{\left(m-m_{o}\right)}{m_{o}}$$

where: *α*–swelling ratio, *g*/*g*; *m*–mass of swollen sample, *g*/*g*; *m_o_*–mass of dry sample (before the study), *g*.

The obtained results are presented in a form of bar charts showing swelling ability of all samples depending on the swelling period and the liquid in which the analysis was performed.

## 4. Conclusions

The performed XRD analysis indicated that two-phase material consisting of hydroxyapatite and tricalcium phosphate was prepared as a result of wet precipitation method. Both phases are classified as calcium phosphates and widely used for bone tissue regeneration.

The analysis of the stability of ceramic phase in PVP solutions at various concentrations allowed to select adequate polymer concentration, i.e., 15%. The sedimentation rate of HAp particles in such a solution was 0.0292 mm/min. Considering the fact that the photopolymerization process leading to the preparation of polymer–ceramic composites takes 120 s, such a sedimentation rate makes the possibility to prepare homogeneous reaction suspensions and thus a proper course of the reaction.

SEM analysis of composites confirmed the presence of the homogeneous layer of the ceramic phase in analyzed materials. Importantly, the presence of the elements characteristic both for polymers used (i.e., PVA and PVP) and for hydroxyapatite was proved. What is more, these results were consistent with the results of FT-IR spectroscopy, where the preparation of materials consisting both of polymers and the ceramic phase was verified (absorption bands characteristic for all these components were observed on obtained FT-IR spectra).

Prepared polymer–ceramic composites showed swelling properties. The highest swelling ratios were calculated for polymer matrices obtained using crosslinker PEGDA 575 swelled in distilled water. It was proved, that the introduction of ceramic phase into the polymer matrices decreased their sorption capability (for example after 1 h of swelling, the swelling ratio for sample 575 was 2.46 *g*/*g* wherein the same property of sample 575_HAp was 1.84 *g*/*g*).

The molecular weight of the crosslinker used during the photopolymerization process affected the surface morphology and the swelling properties of such obtained materials. Samples obtained using crosslinker with lower molecular weight showed greater sorption abilities and their surface was significantly more corrugated compared to the same characteristics of samples prepared using crosslinking agent with higher molecular weight. Thus, it is possible to obtain the material with desirable properties by selecting the crosslinker with specified molecular weight.

Considering such advantages of obtained composites, such as short time of their synthesis, possibility of preparation of materials with desired shapes and sizes as well as their well-developed surface with homogenous hydroxyapatite layer, they seem to be very promising materials with a great application potential in bone tissue regeneration. Moreover, swelling properties seem to be particularly interesting properties of developed composites. During the sorption process, the release of active substances present in such materials may take place. Thus, such materials may also find application in drug delivery systems, and this is the reason why in the future the development of presented investigations toward controlled delivery of drugs reducing the risk of infection during transplantations is planned.

## Figures and Tables

**Table 1:** Data compilation based on ICDD database and Rietveld refinement calculations.

Phase	Space Group	Lattice Parameters	Crystal Size (nm)	Lattice Strain (%)	ICDD Database
					Lattice Parameters
Ca_10_(PO_4_)_6_(OH)_2_ (*HAp*)	P6_3_/m	a = 9.50 Å c = 6.90 Å	7.8	0.63	a = 9.42 Å c = 6.88 Å
Ca_3_(PO_4_)_2_ (*TCP*)	P2_1_/a	a = 12.90 Å b = 27.00 Å c = 15.10 Å β = 127.00°	17.0	0.01	a = 12.89 Å b = 27.28 Å c = 15.22 Å β = 126.2°

**Table 2:** The sedimentation rates of HAp particles in PVP solutions.

Concentration of PVP Solution (%)	Sedimentation Rate (mm/min)
0	0.4405 ± 0.02337
5	0.4243 ± 0.00354
10	0.1329 ± 0.00311
15	0.0292 ± 0.00362
20	0.0161 ± 0.00103

**Table 3:** The compositions of prepared composites.

15% PVP (mL)	5% PVA (mL)	PEGDA 575 (mL)	PEGDA 700 (mL)	Photoinitiator * (mL)	Hydroxyapatite wt. %	Sample
3.0	7.0	2.0	-	0.05	-	575
					10	575_HAp
		-	2.0		-	700
					10	700_HAp

* 2-hydroxy-2-methylpropiophenone, Darocur 1173.

## Data Availability

The data presented in this study are available on request from the corresponding authors.

## Sample Availability

Samples of the compounds are available from the corresponding authors.
